# Surface Modification and Heat Generation of FePt Nanoparticles

**DOI:** 10.3390/ma10020181

**Published:** 2017-02-15

**Authors:** Da-Hua Wei, Ko-Ying Pan, Sheng-Kai Tong

**Affiliations:** Institute of Manufacturing Technology & Department of Mechanical Engineering, National Taipei University of Technology (TAIPEI TECH), Taipei 10608, Taiwan; koyingpan@mail.ntut.edu.tw (K.-Y.P.); k671400@gmail.com (S.-K.T.)

**Keywords:** FePt nanoparticles, surface modification, thiol, heat response, biocompatibility

## Abstract

The chemical reduction of ferric acetylacetonate (Fe(acac)_3_) and platinum acetylacetonate (Pt(acac)_2_) using the polyol solvent of phenyl ether as an agent as well as an effective surfactant has successfully yielded monodispersive FePt nanoparticles (NPs) with a hydrophobic ligand and a size of approximately 3.8 nm. The present FePt NPs synthesized using oleic acid and oleylamine as the stabilizers under identical conditions were achieved with a simple method. The surface modification of FePt NPs by using mercaptoacetic acid (thiol) as a phase transfer reagent through ligand exchange turned the NPs hydrophilic, and the FePt NPs were water-dispersible. The hydrophilic NPs indicated slight agglomeration which was observed by transmission electron microscopy images. The thiol functional group bond to the FePt atoms of the surface was confirmed by Fourier transform infrared spectroscopy (FTIR) spectra. The water-dispersible FePt NPs employed as a heating agent could reach the requirement of biocompatibility and produce a sufficient heat response of 45 °C for magnetically induced hyperthermia in tumor treatment fields.

## 1. Introduction

Nanoparticles (NPs) with magnetic characteristics have been attracting considerable attention due to their wide range of research fields such as high-density information storage media and biomedical potential applications in cell separation, targeted drug delivery, therapy, biological sensing, and magnetic resonance imaging (MRI) [1,2,3,4,5,6,7,8,9,10,11,12,13,14,15]. The ability of magnetic NPs could provide the promising characteristic of control and manipulation by an external magnetic field as an advantage in biomedical assays. The chemically synthesized FePt NPs in organic solvents have been extensively studied, including in many potential applications, due to their attractive electronic, magnetic, optical, catalytic, and biomedical properties. Therefore, the abilities to control the uniform size, chemical stability, and further functionalize the surface of the well-dispersed NPs are very important because all possible potential applications are directly dependent on such properties [16,17,18,19,20,21,22]. The benefit of the organic synthesized method is control over the particle size and shape with close-to–atomic-layer precision, which strongly affects the chemical and physical characteristics of the NPs. As-synthesized metal-based NPs often have hydrophobic chains, causing the NPs to be immiscible in aqueous solutions. For satisfying biological applications, methods must be developed to transfer the organic magnetic NPs into aqueous solutions that could be easily dispersed in blood to exhibit good biocompatibility and directed to a specific target upon applying an external magnetic field [23,24]. The stability and suitability of the magnetic NPs for a certain biological application depend on necessary aspects of the magnetic properties and the surface characteristics. The purpose of this present work is, at first, using a dioctylether and phase transfer reagent to compare the fundamental performance of the chemically synthesized FePt NPs bonded to different surfactant ligands by phase transfer procedures. Secondly, the FePt NPs were transferred into the water state to satisfy the request of biocompatibility via chemical surface modification and they could be easily controlled by a magnetic field to provide enough local heat for magnetic fluid hyperthermia (MFH) applications. Cancer and tumor cells can be killed at temperatures between 42 and 45 °C; furthermore, tumor cells are more sensitive to increases in temperature than normal tissues because they have a weak cooling function. Above 42 °C, cancer cells also become more susceptible to traditional drug treatments [25,26]. Therefore, the magnetic FePt-based NPs can be used as a potential heating agent for applications such as cancerous or tumor hyperthermia.

## 2. Synthesized Procedures of FePt Nanoparticles

All the reagents were used as purchased from commercial sources and without further purification. Briefly, the procedure for synthesizing monodispersive FePt nanoparticles were involved as follows: 0.5 mmol for platinum acetylacetonate Pt(acac)_2_, 1 mmol for iron acetylacetonate Fe(acac)_3_, 3.75 mmol 1,2-hexadecanediol were mixed with 30 mL of phenyl ether. After purging with argon for 30 min at room temperature, the flask was heated up to 100 °C for 30 min with additive 1 mmol oleic acid (C_18_H_34_O_2_) and 0.5 mmol oleylamine (C_18_H_35_NH_2_) stabilizers into flask at the same time. And then the mixture was heated up to 260 °C to reflux phenyl ether for 1 h to form FePt NPs, and the final products were dispersed in hexane. This kind of bottom-up approach to synthesize self-assembled FePt NPs was used in this work [27]. To make these NPs biocompatible in this research work as simulated in human body fluid, the FePt NPs have been transformed in water soluble state by changing the surfactant ligands via mercaptoacetic acid (thiol, C_2_H_4_O_2_S). The crystalline structure and particle sizes were identified by ex situ X-ray diffraction (XRD, PANalytical, Almelo, The Netherlands) and transmission electron microscopy (TEM, JEOL, Tokyo, Japan), respectively. The magnetic properties were characterized by vibrating sample magnetometer (VSM, Lake Shore, Westerville, OH, USA) with the applied field up to 20,000 Oe at room temperature.

## 3. Results and Discussion

**Figure 1:**
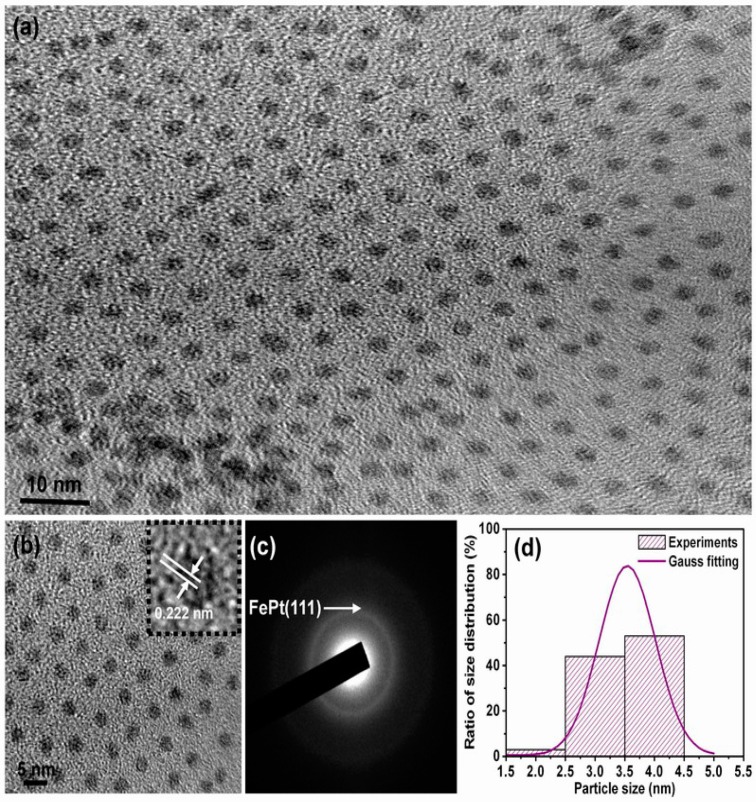
Transmission electron microscopy (TEM) bright field images for the FePt NPs synthesized in phenyl ether capped with oleic acid and oleylamine ligands, respectively. (**a**) Low-magnification image; (**b**) Enlarged magnification of (**a**) and the inset TEM image is high-resolution transmission electron microscopy (HRTEM) of an individual sphere NP; (**c**,**d**) selected area of diffraction (SAD) and particle size distribution with Gauss fitting curve, respectively.

Figure 1 shows the in-plane TEM bright field images and corresponding particle size distribution for the FePt nanocrystals synthesized in phenyl ether. Shown in Figure 1a is a low-magnification TEM image of the as-synthesized monodispersed FePt nanocrystals. The FePt nanocrystals capped with oleic acid and oleylamine ligands could be monodispersed in hydrophobic solvents without significant aggregation and with isolated distances of each nanocrystals, indicating very good crystallinity with a dominant sphere shape. The enlarged magnification of Figure 1a is shown in Figure 1b, and the inset TEM image is a high-resolution transmission electron microscopy (HRTEM) image of an individual sphere nanoparticle (NP). An example of the present FePt NPs synthesized in phenyl ether indicated the perfectly aligned lattice planes as shown in the upper-right inset of Figure 1b, exhibiting a well-crystallized structure. The interplanar spacing of 0.222 nm obtained from the HRTEM image can be ascribed to the adjacent (111) plane of the FePt disordered crystal. The face-centered cubic (fcc) structure feature of the as-synthesized FePt NPs is also shown in their electron diffraction pattern. Figure 1c shows such a pattern from the selected area of diffraction (SAD) of the nanocrystalline assembly. The composition of the NPs was determined to be close to Fe_56_Pt_44_ by nanobeam energy-dispersive X-ray spectroscopy (EDS). The average diameter of the FePt NPs synthesized in phenyl ether was about 3.8 ± 0.34 nm with a narrow size distribution as shown in Figure 1d.

**Figure 2:**
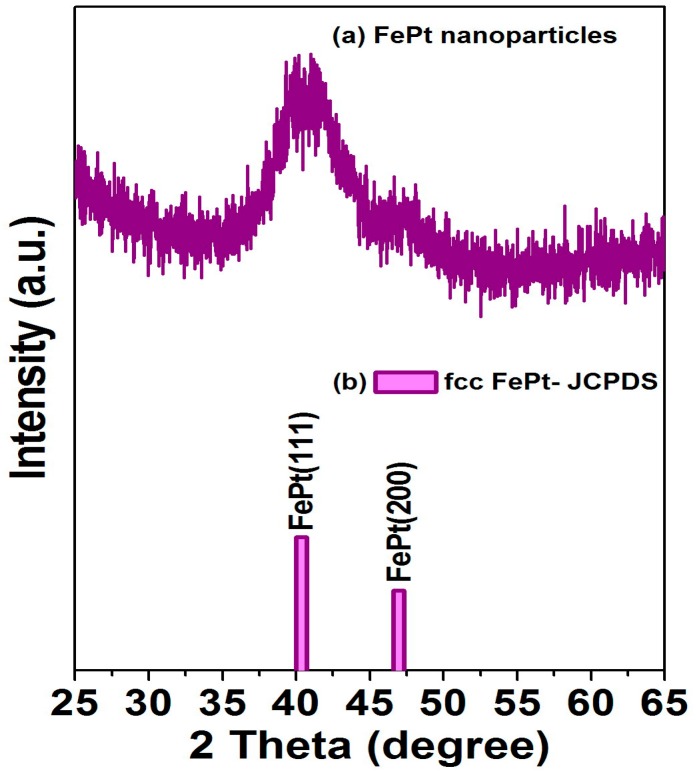
(**a**) X-ray diffraction patterns for the FePt NPs synthesized in phenyl ether; (**b**) diffraction pattern is the corresponding standard Joint Committee on Powder Diffraction Standards (JCPDS) of disordered fcc FePt phase.

The XRD patterns for the FePt nanocrystals synthesized in phenyl ether and the corresponding standard Joint Committee on Powder Diffraction Standards (JCPDS) of the FePt phase are shown in Figure 2a,b, respectively The as-synthesized FePt NPs without any external energy to overcome the activation energy of an ordered phase transformation indicated the formation of the chemically disordered fcc FePt structure with a (111) orientation reacted in phenyl ether. It is clear that the as-synthesized FePt NPs can be indexed to the FePt phase in a cubic structure as shown in Figure 2a. The diffraction peak of the FePt synthesized in phenyl ether was broad, indicating the FePt NPs synthesized in phenyl ether have a small particle size. On the other hand, no diffraction peak of an unclear phase or peak shift of FePt was observed. An average particle diameter of 3.6 nm was calculated from the peak width of the XRD pattern using the Scherer formula, which is consistent with the diameter calculated by the statistical analysis of the TEM images, as shown in Figure 1. The above structural characterizations show clearly that fcc FePt NPs with a composition of Fe_56_Pt_44_ were synthesized by the co-reduction of Fe(acac)_3_ and Pt(acac)_2_ in the presence of 1,2-hexadecanediol (HDD) in phenyl ether. More importantly, the HDD also played a role as an effective surfactant. Based on structure and functionality, using HDD as a surfactant for dispersing the formed NPs is not unreasonable, and it also provides the reductive species needed to form the fcc FePt NPs. In addition, it is possible that the oxidized HDD molecules are also used as capping ligands to protect the NPs from oxidation and to facilitate the dispersion of NPs in nonpolar solvents. Traditional methods such as capping ligands oleic acid and oleylamine are used as the surfactants in a proper solvent.

**Figure 3:**
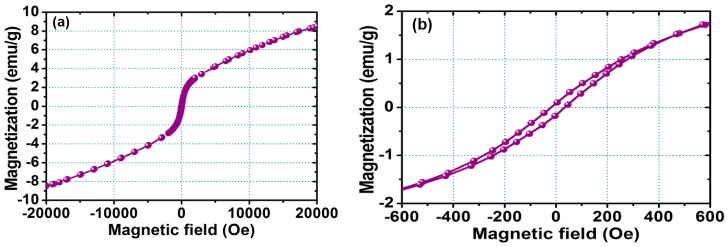
(**a**) Magnetization loop measured at room temperature for the FePt NPs measured at room temperature; (**b**) corresponding enlarged hysteresis loop for FePt NPs ranging in the field of ±600 Oe.

Figure 3a shows the VSM hysteresis loop for the FePt NPs measured at room temperature, and Figure 3b shows the corresponding enlarged hysteresis loop for FePt NPs ranging in the field of ±600 Oe, respectively. The hysteresis loops are superparamagnetic at room temperature as shown in Figure 3. The magnetization value of FePt NPs in a field of 20,000 Oe was 8.4 emu/g, with a coercive force of 30 Oe, while the synthesized solvent was phenyl ether. The magnetic character of FePt NPs was not only strongly correlated with the degree of the chemical ordered phase transformation, but it was also affected by the stoichiometic composition and the bonding of their surface ligands.

**Figure 4:**
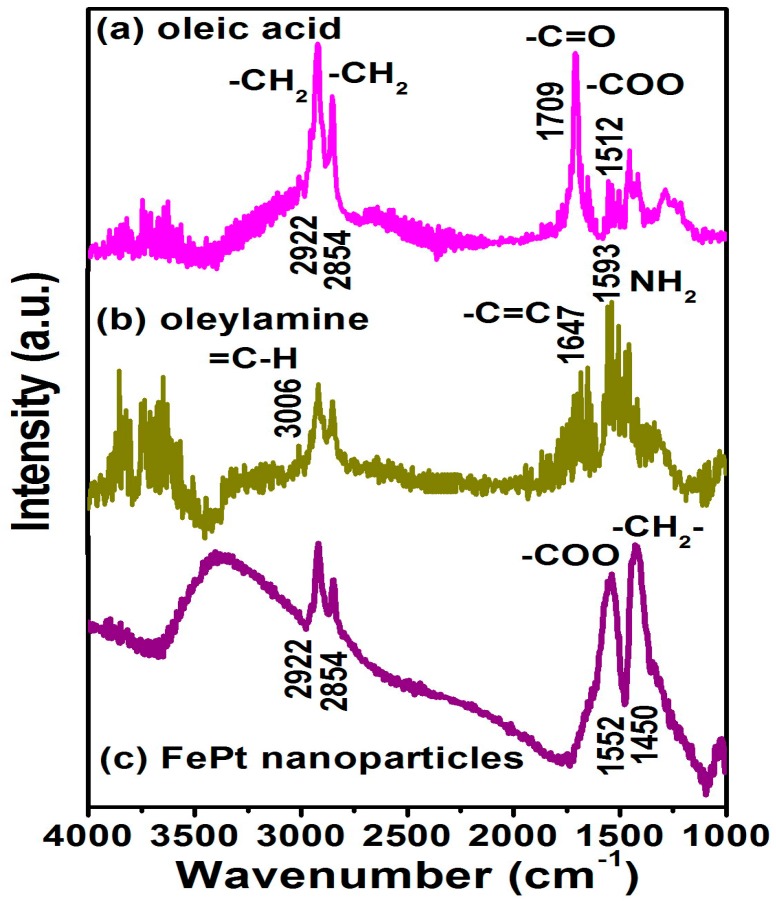
Fourier transform infrared spectroscopy (FTIR) spectra are for the pure (**a**) oleic acid and (**b**) oleylamine, respectively; (**c**) FTIR spectra for the FePt NPs synthesized in phenyl ether capped with oleic acid and oleylamine, respectively.

Figure 4 shows the Fourier transform infrared spectroscopy (FTIR) spectra for the FePt NPs synthesized in phenyl ether capped with oleic acid and oleylamine, respectively. Figure 4a,b are the reference spectra for pure oleic acid and oleylamine, respectively. All spectra in Figure 4 reveal the characteristic peaks of the oleyl group: the peaks at 2854 and 2922 cm^−1^ were due to the symmetric and asymmetric CH_2_ stretching modes. On the other hand, the peak of the FePt NPs at 1552 cm^−1^ was due to the bidendate COO mode of oleic acid binding as shown in Figure 4c. The characteristic peak at 1709 cm^−1^ in the oleic acid spectrum was due to the vibrational ν(C=O) monodendate mode, and the 1512 cm^−1^ peak was due to the bidendate COO mode in the oleic acid. The characteristic peak at 3006 cm^−1^ in the oleylamine spectrum was due to the ν(C–H) mode of the C–H bond adjacent to the C=C bond, and the small peak at 1647 cm^−1^ was due to the ν(C=C) stretch mode. The 1593 cm^−1^ peak was due to the NH_2_ scissoring mode, which suggests that oleylamine was adsorbed with the NH_2_ group intact. Figure 4c indicates the oleic acid and oleylamine both bonded to the as-made FePt NPs synthesized in phenyl ether, thus causing the presence of bidentate carboxylate bonding to the as-synthesized NPs. The observation of both peaks at 1552 and 1450 cm^−1^ was due to the vibrational ν(COO) and stretching (CH_2_) modes, indicating that oleic acid and oleylamine complex surfactants bond to the FePt NPs in both monodentate and bidentate types [28]. 

**Figure 5:**
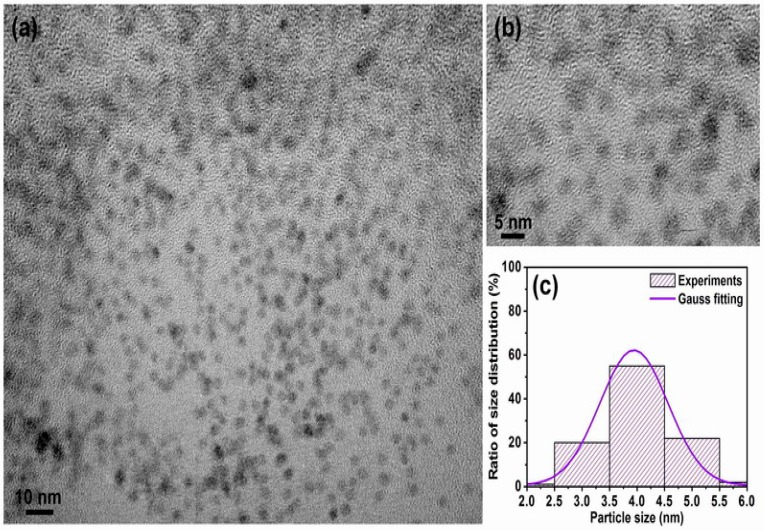
TEM bright field images for the FePt NPs synthesized in phenyl ether after ligand exchange with thiol. (**a**) Low-magnification image; (**b**) enlarged magnification of (a); (**c**) corresponding particle size distribution with Gauss fitting curve, respectively.

Figure 5 shows the in-plane TEM bright field images for the FePt NPs synthesized in phenyl ether after ligand exchange with thiol. Shown in Figure 5a is a low-magnification TEM image of the monodispersive FePt NPs ligands exchanged with thiol, and shown in Figure 5b is the enlarged magnification of Figure 5a. The average diameter of thiol-treated FePt NPs synthesized in phenyl ether was about 4.0 ± 0.38 nm with a narrow size distribution as shown in Figure 5c. Compared with the TEM images of the oleic acid- and oleylamine-capped FePt NPs, it can be observed that the FePt NPs dispersed in hexane tend to self-assemble, maintaining a regular distance between them as shown in Figure 1, whereas in the case of thiol-exchanged FePt NPs, there is no sign of a pattern, indicating that the FePt NPs slightly agglomerated with a smaller interparticle distance after the ligand exchange with thiol. This aggregation effect was due to the shorter chain length of mercaptoacetic acid (C_2_H_4_O_2_S) compared to that of oleic acid (C_18_H_34_O_2_) or oleylamine (C_18_H_35_NH_2_) stabilizers. 

**Figure 6:**
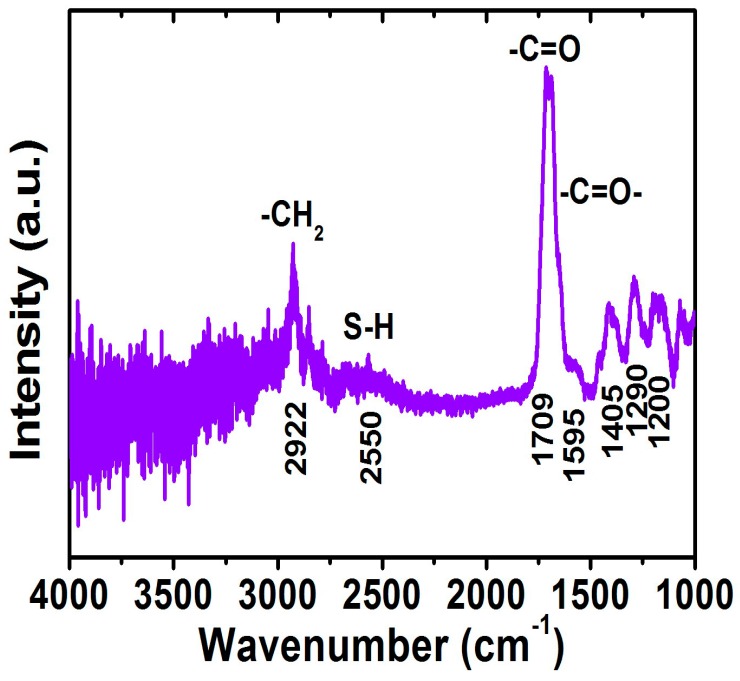
FTIR spectrum for the FePt NPs after ligand exchange with thiol.

The FTIR spectrum for the FePt NPs after the ligand exchange with thiol was shown in Figure 6. For the FePt NPs that underwent a ligand exchange with thiol, the disappearance peak and decreased intensity peak of CH_2_ stretching modes were at 2854 and 2922 cm^−1^, indicating complete changes in the surface chemistry of the FePt NPs as shown in Figure 6. The peaks at 1709 and 1595 cm^−1^ were observed due to the C=O stretch vibration mode of alkyl thiol chains as well as weaker absorption peaks at 1405, 1290, and 1200 cm^−1^ as shown in Figure 6. The absorption peak at 2550 cm^−1^ derived from the S–H stretching vibration of the thiol chains can be seen in the FTIR spectra of the FePt-thiol NPs, indicating the interactions between FePt NPs and thiol chains [29]. Actually, the FTIR spectra for the FePt NPs before and after ligand exchange were indeed different when compared with the as-synthesized NPs in phenyl ether, as shown in Figure 4c and Figure 6, which confirms the thiol functional group (SH) bonded to the Fe or Pt surface because of their weak absorption of Pt–S or Fe–S bonds located at low wavenumbers [30]. The above evidence showed that the oleic acid and oleylamine ligands were replaced by the SH functional group, and the reagent that contained the thiol functional group was easily approached to replace the surfactant on the definite metal surface which also confirms that thiol bonds to the surface of the FePt NPs. In order to confirm that the FePt NPs could be dispersed in the water phase, as simulated in human blood, and provide heat for hyperthermia, this analysis means that the FePt-based NPs could stable in a water solution [31]. 

**Figure 7:**
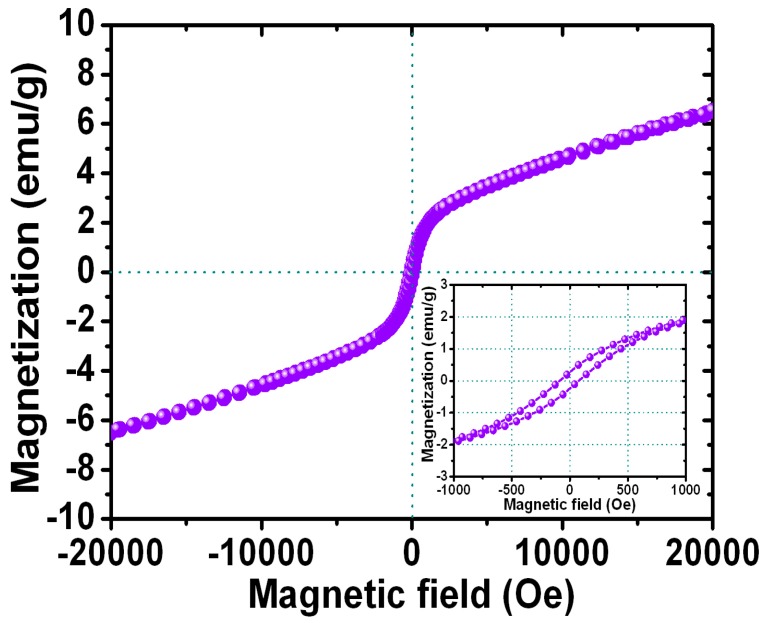
Magnetization loop for the FePt NPs after ligand exchange with thiol. The inset shows the corresponding enlarged hysteresis loops for FePt-thiol NPs measured in the field of ±1000 Oe.

Figure 7 shows the magnetization loops for the FePt NPs after the ligand exchange with thiol. The inset shows the corresponding enlarged hysteresis loops for FePt-thiol NPs ranging in the field of ±1000 Oe. The hysteresis loop for the FePt-thiol NPs was also superparamagnetic at room temperature as shown in Figure 7. The magnetization value of FePt NPs in the field of 20,000 Oe was 6.5 emu/g and with a coercive force of 58 Oe while the synthesized solvent was phenyl ether and then ligand-exchanged with thiol. The magnitude of the magnetization for FePt-thiol was smaller than that of FePt NPs. The maximum magnetization values were 8.4 emu/g and 6.5 emu/g for FePt and FePt-thiol NPs, respectively. The magnetization values of FePt-thiol in all fields were lower than that of pure FePt NPs. The changed magnetization after the ligand exchange with thiol was due to the change in the dipole moment. From the enlarged hysteresis loops, we can identify another important difference in the coercivities between FePt and FePt-thiol NPs. The coercivity values were 30 and 58 Oe for FePt and FePt-thiol NPs, respectively. The coercivity showed a clear increase for FePt-thiol NPs. This observation likely reflects the fact that the coercivity of a weakly ferromagnetic NP is related to the particle size [32]. Therefore, the increase in the coercivity can be attributed to the larger size of FePt-thiol NPs which leads to a less-effective coupling of the magnetic dipole moments. So this presents FePt-thiol–based NPs with a suitable size and magnetic characteristics that could be easily controlled by applying an external magnetic field for potential biomedical applications. 

**Figure 8:**
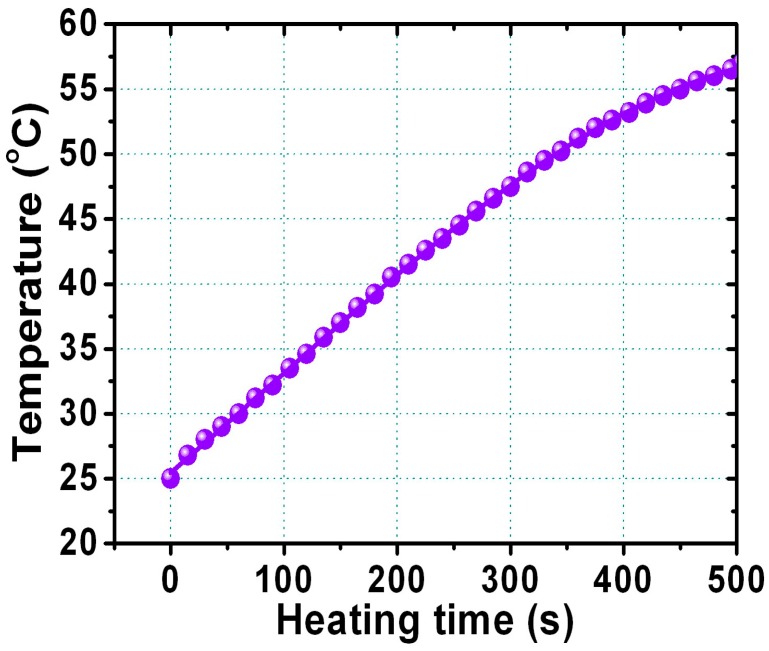
The relationship between rising temperature and the heating time for FePt NPs dispersed in water after ligand exchange with thiol.

Finally, the heating response of hydrophilic FePt NPs was measured to prove the self-heating temperature-increasing characteristics for use as an in vivo hyperthermia agent in biomedicine. Figure 8 shows the rising temperature as a function of the heating time dispersed in water for the FePt NPs after the ligand exchange with thiol. For the dispersed liquid FePt NPs, the temperature of the liquid was measured with a noncontact temperature sensor using an optical fiber thermometer. The hydrophilic FePt NPs that underwent a ligand exchange with thiol could produce enough local heat (at least 45 °C) to kill cancerous and tumor cells for potential heating therapy treatments. The amount of heat energy generated by magnetic FePt NPs can be determined by the applied magnetic field, magnetization, frequency, and particle volume because the heat energy generation is described by power loss due to Brownian and Néel relaxation mechanisms [33,34,35]. Our present hydrophilic FePt NPs with a maximum specific absorption rate (SAR) value of 20 W/g were obtained at 700 kHz and a 3.8 kAm^−1^ alternating current (AC) magnetic field for FePt NPs with a concentration of 1 mg·mL^−1^. These hydrophilic FePt-based NPs with good biocompatibility can be used as a potential carrier and as thermoseeds in biological applications.

## 4. Conclusions

First, monodispersive FePt magnetic nanoparticles (NPs) with hydrophobic ligands were chemically synthesized, and then were developed with tunable surface-functional properties using a phase transfer reagent in the present work. The water-soluble FePt NPs are generally considered to be biocompatible via ligand exchange by thiol. The monodispersive and hydrophilic FePt NPs could produce enough local heat to damage proteins and structures within cancerous or tumor cells for disease therapy using high-frequency waves outside the body. It is believed that such hydrophilic FePt-thiol NPs can provide a possible application for magnetic hyperthermia treatments of cancer or tumors. These hydrophilic FeP NPs can also potentially be used as a nanomedicine or for molecular targeting by nano-engineered devices.
